# Regenerative Strategies for Vocal Fold Repair Using Injectable Materials

**DOI:** 10.3390/biomimetics10110748

**Published:** 2025-11-06

**Authors:** Se Hyun Yeou, Yoo Seob Shin

**Affiliations:** Department of Otorhinolaryngology-Head and Neck Surgery, Ajou University School of Medicine, Suwon 16499, Republic of Korea; scieth17@ajou.ac.kr

**Keywords:** injectable biomaterials, vocal fold regeneration, hydrogel, extracellular matrix remodeling

## Abstract

Injectable biomaterials for vocal fold disorders are being developed to provide not only mechanical reinforcement but also a regenerative microenvironment. Recent hydrogels based on hyaluronic acid (HA) derivatives, calcium hydroxylapatite and decellularized matrix scaffolds are designed to approximate the viscoelastic behavior of native tissue, allow controlled degradation, and modulate local immune responses. Rather than serving merely as space-filling agents, several of these materials deliver extracellular matrix (ECM)-like biochemical signals that help maintain pliability and overcome some limitations of conventional augmentation. Experimental and early clinical studies involving growth factor delivery, stem cell-based injections, and ECM-mimetic hydrogels have demonstrated improved mucosal wave vibration and reduced fibrosis in cases of scarring. In clinical series, benefits from basic fibroblast growth factor can persist for up to 12 months. Further progress will depend on correlating material properties with objective vibratory performance to achieve lasting restoration of phonation and advance true tissue-regenerative therapy.

## 1. Introduction

Vocal fold injection has become a widely adopted therapeutic modality for a spectrum of laryngeal disorders characterized by glottic insufficiency. The technique was first introduced in the mid-20th century as a method of medializing the vocal fold, and it has since evolved into a versatile intervention applicable to unilateral vocal fold paralysis or paresis, presbylaryngis, benign atrophy, sulcus and scarring, and selected cases of glottic gap from structural or iatrogenic causes [1]. The underlying principle is to restore glottic competence by improving closure during phonation, thereby reducing breathiness, improving voice quality, and preventing aspiration.

Over time, the objectives of injection therapy have expanded beyond simple medialization. In vocal fold paralysis, augmentation primarily provides bulk to compensate for glottic gap and improve voice and airway protection. In presbylaryngis and age-related atrophy, injectables restore lost volume and tension of the thyroarytenoid muscle. In vocal fold scarring or sulcus, the therapeutic challenge lies not only in augmenting volume but also in recreating the viscoelastic properties of the lamina propria, which are essential for efficient mucosal wave vibration [2,3]. Thus, injectables are increasingly viewed not merely as passive fillers but also as biomaterials capable of modulating the microenvironment, delivering bioactive factors, and promoting regeneration.

Parallel to these clinical applications, there has been remarkable progress in material science. Early agents such as paraffin, silicone, and Teflon™ were eventually abandoned due to granulomatous reactions and long-term morbidity [4]. Current practice utilizes a range of temporary injectables (e.g., hyaluronic acid (HA), carboxymethylcellulose) and longer-lasting agents (e.g., calcium hydroxylapatite, autologous fat), each selected based on the patient’s diagnosis, expected disease course, and therapeutic goals [5,6]. Moreover, next-generation biomaterials such as silk-HA composites and peptide-based hydrogels are being developed with dual aims: providing biomechanical support and actively engaging in tissue regeneration [7,8].

In this context, injectable biomaterials should be considered as part of a broader therapeutic continuum: from short-term diagnostic or bridging interventions to long-term augmentation strategies and regenerative therapies. This review will therefore summarize the structural and pathophysiologic basis of the vocal fold, outline the principles of injectable materials, and discuss their clinical and regenerative applications across vocal fold paralysis, presbylaryngis, and scarring, with emphasis on both historical perspectives and recent innovations.

## 2. Vocal Fold Structure and Pathophysiology

### 2.1. Normal Structure, Development, and Aging of the Vocal Fold

**Figure 1:**
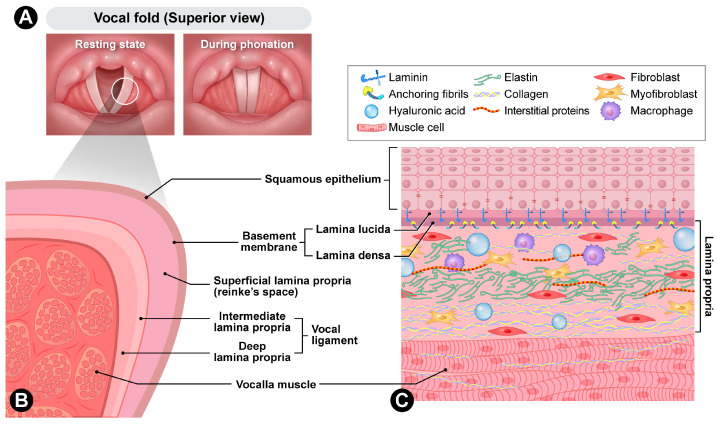
Anatomy of vocal folds. (**A**) Superior view of resting state and during phonation. (**B**) Cross-section. (**C**) Cellular composition.

The vocal folds are located within the larynx and primarily function to protect the airway, with a secondary role in voice production. Movement of the arytenoid cartilages facilitates the adduction and abduction of the vocal folds, enabling them to close during phonation and open during respiration (Figure 1). During sound production, the vocal folds oscillate in a wave-like pattern, a phenomenon made possible by their highly specialized, multilayered structure, which provides remarkable mechanical adaptability [9,10,11].

The human vocal fold is a highly specialized vibratory tissue whose layered microanatomy and extracellular matrix (ECM) composition enable efficient sound production. The surface stratified squamous epithelium forms a protective barrier connected to a basement membrane zone (BMZ), which provides mechanical coupling to the underlying lamina propria. The BMZ contains type IV collagen, laminin, and collagenous anchoring structures that link basal epithelial cells to the superficial lamina propria (SLP). Anchoring fibers composed of type VII collagen extend from the lamina densa into the SLP, ensuring continuity between the epithelium and stroma [12].

Beneath the BMZ, the lamina propria is traditionally divided into three layers: superficial, intermediate, and deep [13]. The SLP, also known as Reinke’s space, is rich in HA and proteoglycans and contains a sparse and loosely organized fibrillar network. This composition facilitates shear displacement and the propagation of the mucosal wave. The intermediate layer contains more organized elastin fibers, while the deep layer consists of densely packed type I collagen [14]. Together, the intermediate and deep layers form the vocal ligament. The underlying thyroarytenoid (vocalis) muscle modulates tension and bulk, allowing fine control of pitch and glottic closure.

From a materials science perspective, the vocal fold can be considered an anisotropic soft tissue. Its anisotropy results from the predominant alignment of collagen and elastic fibers along the anterior–posterior axis, which imparts direction-dependent mechanical properties [15]. Specifically, the tissue is significantly stiffer in the longitudinal direction while exhibiting greater compliance transversely [16]. This directional dependence is not merely a histological observation but a fundamental biomechanical characteristic that supports high-frequency oscillation and ensures both efficiency and stability during phonation [17].

Another defining characteristic of the vocal fold is its nonlinear mechanical response. At low strains, the tissue remains relatively compliant; however, beyond a certain threshold, stiffness increases disproportionately, producing substantial resistance to further deformation [16]. This behavior results from the recruitment and straightening of collagen fibers, which are initially arranged in a wavy configuration. Through this mechanism, the vocal fold maintains high sensitivity to the small forces required for oscillation while simultaneously resisting overstretching that could cause structural damage [17]. Therefore, nonlinearity provides an essential balance between vibratory efficiency and mechanical protection.

Nevertheless, the vibratory behavior of the vocal folds at higher frequencies cannot be fully explained by nonlinear elasticity alone. Experimental data demonstrate that tissue stiffness increases significantly with frequency compared to static conditions [18], and both viscosity and elasticity exhibit frequency-dependent variations [19]. Furthermore, nonlinear viscoelastic effects become more pronounced under large strains, while interactions among collagen fibers, the ECM, and interstitial fluid contribute additional damping and energy dissipation [20]. For these reasons, accurate characterization of high-frequency vocal fold vibration requires constitutive models that incorporate frequency-dependent viscoelasticity alongside microstructural interactions.

The layered organization of the lamina propria is absent at birth. Pediatric histology shows that the lamina propria is initially undifferentiated and later develops into a tri-laminar structure. Differences in cell density become evident within the first few months of life, followed by progressive deposition and alignment of collagen and elastin fibers throughout childhood. By adolescence, the tri-laminar organization is fully established, providing the biomechanical specialization necessary for mature voice production [21].

With aging, remodeling of the ECM alters vibratory capacity. Histological analyses indicate age-related changes in collagen abundance and organization, elastin integrity, and levels of small leucine-rich proteoglycans that regulate fibrillogenesis. These changes affect tissue stiffness and disrupt the viscoelastic balance [22]. Both animal and human studies demonstrate denser collagen bundles and decreased elastin fibers in aged tissue, while HA content also tends to decline [23]. These alterations clinically contribute to glottic bowing, increased phonation threshold, and presbyphonia [24].

### 2.2. Pathophysiology of Vocal Fold Disease

Disruption of the vocal fold’s layered microarchitecture impairs viscoelastic tuning and glottic closure, resulting in dysphonia and, in some cases, aspiration. Pathology may primarily affect the ECM (e.g., scarring, sulcus), epithelial–BMZ integrity (e.g., nodules), neuromuscular control (e.g., paralysis), or a combination of these domains in oncologic and treatment-related disease [13,25].

#### 2.2.1. Vocal Fold Scarring

Chronic scarring is a prototypical form of pathologic ECM remodeling characterized by increased collagen bundles, fragmented or disorganized elastin, and reduced HA content. In a well-established rabbit model evaluated six months after injury, the scarred lamina propria exhibited a higher elastic shear modulus and increased viscosity compared to controls [26]. These findings are consistent with stiffer and more viscous tissue that dampens mucosal wave amplitude. Human histology similarly demonstrates excessive collagen deposition and ECM disarray as hallmarks of chronic scarring [27].

#### 2.2.2. Sulcus Vocalis

Sulcus vocalis is characterized by thinning or loss of the SLP. Electron microscopy reveals that the sulcus is confined to the squamous epithelium, with basement membrane thickening and thinning of the SLP at the sulcus base, where collagen fibers are dense and elastic fibers are decreased [28]. These structural deficits tether the epithelial layer to deeper tissues, reducing vibratory capacity and resulting in impaired wave propagation, persistent hoarseness, and a restricted pitch range [29,30].

#### 2.2.3. Benign Vocal Fold Lesions

Benign lesions of the vocal folds include nodules, polyps, and cysts, each representing a localized disturbance of ECM organization. Electron microscopy of phonotraumatic lesions has revealed irregularities of the basement membrane and, in some cases, disrupted epithelial junctions. These alterations may weaken epithelial anchoring and impair vibratory efficiency.

Vocal nodules are typically associated with chronic phonotrauma and show thickening of the basement membrane, epithelial hyperplasia, and fibroblast proliferation [31].

Vocal polyps are typically unilateral and develop at the midpoint of the membranous vocal fold, where shear stress is maximal during phonation [32]. Histologically, they present with focal accumulation of edematous matrix and dilated capillaries within the SLP, sometimes accompanied by fibrin exudation or partial duplication of the basement membrane. The localized increase in mass and stiffness alters body–cover dynamics, resulting in asymmetric vibration and a breathy or rough voice quality [31]. Unlike vocal nodules, polyps usually lack epithelial hyperplasia and tend to arise acutely after phonotrauma or subepithelial hemorrhage rather than from chronic mechanical stress [33]. The ensuing stromal edema and vascular congestion further disrupt mucosal-wave propagation [25].

Vocal fold cysts may emerge in association with upper respiratory tract inflammation or phonatory strain, and chronic vibratory trauma is regarded as a key etiologic contributor to their development [34]. Vocal fold cysts typically occur on one side and may induce reactive edema or a contact lesion on the opposite fold. Vocal fold cysts are subclassified as epidermoid and mucous retention types. The epidermoid cyst is lined by keratinizing epithelium and enclosed by a fibrous capsule, whereas the mucous retention cyst arises from ductal obstruction and retains a thin glandular wall. Both forms create focal stiffness within the vibratory portion of the fold, reducing local pliability and limiting vibratory amplitude [35].

#### 2.2.4. Malignant Vocal Fold Lesions and Treatment-Related Status

Laryngeal squamous cell carcinoma disrupts the normal layered architecture of the vocal fold, invading the lamina propria and breaching the basement membrane zone [36]. Subsequent oncologic treatments further aggravate tissue injury through microvascular compromise, ischemia, and persistent inflammation. After radiotherapy, the vocal fold mucosa typically shows excessive collagen deposition, activation of profibrotic pathways such as TGF-β signaling, and compositional changes in other ECM components [37]. Comparable remodeling, including fibronectin accumulation, has been consistently demonstrated in experimental models [38]. Rodent studies support these observations, revealing collagen and fibronectin deposition accompanied by TGF-β upregulation and fibroblast activation within the lamina propria [38,39].

#### 2.2.5. Vocal Fold Paralysis

Unilateral or bilateral vocal fold paralysis and paresis represent neuromuscular, rather than ECM pathology. The primary defect is impaired adduction, which leads to glottic insufficiency, breathy dysphonia, and aspiration. Following recurrent laryngeal nerve injury, denervation of the intrinsic laryngeal muscles produces flaccidity and lateral bowing, and chronic cases may exhibit partial reinnervation or synkinetic miswiring that causes uncoordinated contraction [40,41]. Chronic denervation also causes muscle atrophy and secondary remodeling of the surrounding connective tissue. Management in such cases emphasizes bulk restoration and medialization rather than ECM replacement [32].

#### 2.2.6. Vocal Fold Atrophy

Vocal fold atrophy is defined by loss of thyroarytenoid muscle fibers and thinning of the lamina propria [42]. Aging, disuse, and chronic denervation contribute to reduced muscle volume and may be accompanied by decreased capillary density, increased interstitial collagen deposition, and diminished levels of elastin and HA. These alterations disrupt the viscoelastic balance of the vocal fold cover, leading to diminished mucosal wave amplitude and incomplete glottic closure. Histologically, muscle fibers exhibit a smaller cross-sectional area with occasional fatty infiltration, and the ECM shows disorganization of collagen and elastin fibers [43,44]. The resulting disparity between muscular and stromal components gives rise to a bowed, flaccid vocal fold that manifests as glottic insufficiency.

## 3. Principles of Injectable Materials

### 3.1. Mechanical and Rheological Design

Matching the mechanical behavior of injectable materials to that of the native vocal fold lamina propria is a critical design requirement for phonatory restoration. The viscoelastic properties of the injected material, including its elastic modulus, viscosity, and stress-relaxation behavior, should approximate those of normal vocal fold tissue to maintain smooth mucosal wave propagation. Experimental studies have shown that the human vocal fold mucosa exhibits very low stiffness, with a shear modulus on the order of 10^2^ to 10^3^ Pa [45]. In contrast, whole vocal fold samples that include the ligament layer display anisotropic behavior, with a Young’s modulus of approximately 1 kPa in the transverse direction and about 30 kPa longitudinally, along with a highly nonlinear stress–strain profile in which the slope increases 10 to 15 times from low- to high-strain conditions [16]. These findings indicate a depth-dependent stiffness gradient across the lamina propria, increasing toward the vocal ligament. Materials that fall well outside this physiological range may compromise pliability or vibratory stability. Excessively stiff fillers can restrict mucosal motion, whereas overly compliant matrices may dissipate vibratory energy too rapidly. The ability of a material to relax stress under constant strain is also important because it governs how vibratory energy is absorbed and released during oscillation. Gaston et al. demonstrated that HA hydrogels with balanced viscoelasticity supported tissue repair and preserved pliability in a rabbit vocal fold model [46]. Hydrogels with appropriately tuned viscous and elastic components therefore appear more capable of maintaining physiological energy storage and damping during phonation.

Injectability focuses on the practical manipulation of materials that already meet mechanical compatibility requirements. The material should allow smooth syringe-based delivery through fine needles and recover its viscosity after injection. Shear-thinning or thixotropic behavior facilitates easy extrusion under pressure and viscosity recovery in situ, which helps achieve uniform placement with minimal injection force [47]. Ease of manipulation depends on predictable flow under constant pressure and resistance to clogging during injection. After placement, controlled crosslinking and polymer concentration are necessary to maintain shape fidelity and volume retention while preventing migration or early loss [48,49]. Appropriate rheological control, therefore, serves a practical rather than mechanical function, ensuring consistent handling and stability during clinical use.

### 3.2. Biological and Immunological Compatibility

Recreating the biochemical and structural features of the native ECM is a fundamental principle in designing biomaterials for vocal fold repair. Native ECM components such as collagen, elastin, and HA determine the viscoelastic and biological properties of the lamina propria, and their functional analogs have been widely used to replicate these characteristics [50]. Incorporating bioactive motifs, including adhesive peptides or growth factor–binding domains, can further enhance fibroblast adhesion and promote controlled matrix remodeling [51]. Achieving this level of biochemical fidelity enables injectable systems to provide an instructive microenvironment that supports organized ECM deposition and maintains tissue pliability throughout the healing process.

Equally important to the biochemical design of a biomaterial is its immunological compatibility. Uncontrolled inflammation or fibrotic encapsulation can disrupt the ECM and impair vocal fold vibration. To prevent such adverse reactions, biomaterials are formulated to minimize the release of inflammatory mediators and to eliminate residual impurities or endotoxins during preparation. Increasing evidence also highlights macrophage polarization as a key determinant of healing: materials that promote an M2-dominant, anti-inflammatory response tend to support tissue integration and immune tolerance, whereas unmodified synthetic scaffolds often sustain a pro-inflammatory M1 state [51,52]. When biochemical mimicry is combined with appropriate immune modulation, the resulting microenvironment favors functional regeneration of the vocal fold over fibrotic repair.

### 3.3. Degradation and Long-Term Performance

After the early healing phase, the long-term stability of an implanted material and the regulation of its degradation become critical factors in maintaining phonatory function. The material must remain in place long enough to support matrix remodeling and sustain augmentation, yet degrade in a predictable way to avoid chronic inflammation or foreign-body reactions. Degradation behavior is especially important because bioactive materials cannot provide lasting augmentation if they disintegrate before tissue remodeling is complete [53]. Maintaining a balance between durability and turnover supports the gradual transfer of mechanical function from the material to the newly formed ECM, consistent with the broader principle that scaffold degradation should preserve structural guidance while enabling tissue integration [51]. In the vocal fold, degradation kinetics must be carefully tuned: premature resorption can cause recurrent insufficiency, whereas excessive persistence may result in stiffness and reduced vibration. Biodegradable polymers such as HA, polyethylene glycol, and polyesters can be engineered to show adjustable degradation rates through control of crosslinking density and polymer chemistry [53]. Moreover, degradation byproducts should be non-toxic and readily eliminated from the tissue microenvironment to prevent secondary inflammation or fibrosis, as highlighted in recent immunological studies of vocal fold biomaterials [51]. When degradation behavior is appropriately controlled, injectable materials can achieve both structural restoration and long-term integration in harmony with the dynamic remodeling of vocal fold tissue.

**Figure 2:**
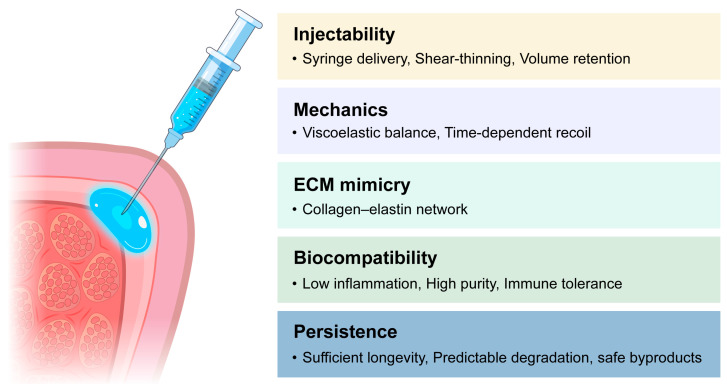
Design criteria for vocal fold injectable materials.

Furthermore, the material should be easily removable in the event of malposition or overcorrection, allowing clinicians to reverse unwanted augmentation without damaging the surrounding tissue [54] (Figure 2).

## 4. Injectable Materials for Vocal Fold Palsy; Volume Augmentation

### 4.1. Conventional Materials

Vocal fold injection was first introduced by Bruening in 1911, who used paraffin to treat unilateral vocal fold paralysis [55]. During the mid-20th century, various materials including paraffin, silicone (polydimethylsiloxane, PDMS), and Teflon™ (polytetrafluoroethylene paste) were employed. However, severe foreign-body reactions, fibrosis, and granuloma formation led to their eventual abandonment [56]. Since then, clinicians have sought injectable substances that can replicate the viscoelastic properties of the native lamina propria while maintaining biocompatibility and controllable persistence.

Following Teflon, collagen emerged as one of the most notable materials for vocal fold augmentation. As a principal component of the vocal fold lamina propria, collagen offers low immunogenicity and excellent biocompatibility. Ford et al. (1984) were the first to introduce bovine collagen for this purpose; however, early formulations carried risks of viral transmission and hypersensitivity reactions [57]. Subsequently, autologous collagen was developed, but the need for donor-site harvesting, complex processing, and high costs limited its clinical application. To address these drawbacks, homologous collagen derived from cadaveric human dermis (Cymetra^®^) was introduced. Cymetra^®^ provided temporary benefits for glottic insufficiency, lasting approximately two to three months, but its use has largely declined with the advent of HA-based injectables [58,59].

Autologous fat injection was first reported by Dedo in 1983, with the injection technique later refined by Mikaelian et al. in 1991 [60]. Fat grafting offers biocompatibility and viscoelastic properties similar to native tissue; however it requires tissue harvesting under general anesthesia and exhibits unpredictable resorption, often necessitating an overcorrection of approximately 30% [61]. Autologous fascia, introduced by Rihkanen in 1998, has the advantages of low absorption and high collagen content, but its lower viscoelasticity and the need for deeper donor incisions remain disadvantages [62].

During the 1990s, safer and more predictable temporary injectable materials were developed. Gelatin (Gelfoam^®^) provided an effect lasting approximately 4–6 weeks and was well tolerated; however, its low viscoelasticity and short duration have limited its current use [63]. HA, a native ECM component of the lamina propria, became the most widely adopted temporary filler due to its excellent biocompatibility and rheological similarity to natural tissue. HA effectively alleviates breathy dysphonia and aspiration symptoms in unilateral vocal fold paralysis [59,64]. Because native HA is rapidly degraded, cross-linked formulations were developed to provide mechanical stability, maintaining physical presence for 4–6 months and clinical benefit for up to 12 months [65]. Current products include Hylaform^®^ (avian-derived), Restylane^®^ (bacterially fermented), and Reviderm^®^, the latter incorporating dextran microspheres (40–60 μm) to slow absorption [59].

To achieve longer-lasting results, calcium hydroxyapatite (CaHA), a naturally occurring component of bone and teeth, was introduced. Among currently approved materials, it is the only injectable substance officially recognized by the U.S. Food and Drug Administration (FDA) for permanent vocal fold augmentation. The material demonstrates excellent biocompatibility and has shown no significant risk of granuloma formation, inflammatory reaction, hypersensitivity, or infection in clinical use [66,67]. Radiesse^®^, composed of CaHA microspheres suspended in a carboxymethylcellulose (CMC) carrier gel, remains the only FDA-approved long-term injectable for vocal fold augmentation. The reported duration of effect averages 18 months, with persistence of up to two years in some cases [68]. The CMC carrier is also used independently as a temporary filler (Radiesse Voice Gel™), providing effects lasting 2–3 months [69].

Overall, contemporary injectable materials exhibit enhanced biocompatibility and mechanical stability compared to earlier agents. However, they primarily function as volumetric fillers with limited ability to restore viscoelastic properties or promote ECM remodeling. These limitations have driven ongoing research into next-generation bioactive and regenerative injectables designed to provide not only mechanical augmentation but also functional tissue repair, representing a promising future direction in voice restoration therapy. A comparative summary of injectable materials used for vocal fold augmentation is presented in Table 1 [53,54,69].

### 4.2. New Synthetic Filler and Approach

Subsequent decades witnessed the introduction of synthetic polymer-based fillers, including polyacrylamide hydrogel (PAAG), polymethylmethacrylate (PMMA), and polydimethylsiloxane (PDMS). PAAG is a hydrophilic gel composed of more than 97% water, offering stable augmentation with minimal resorption, although rare cases of granuloma formation have been reported [70,71]. PMMA consists of nonresorbable microspheres suspended in a collagen carrier that stimulates fibroblast ingrowth, resulting in long-lasting volume maintenance [72]. The representative product Artecoll^®^, containing 20% PMMA microspheres dispersed in 80% bovine collagen, achieves durable augmentation as the carrier degrades within three months and host fibroblasts infiltrate the interparticle spaces. In patients with unilateral vocal fold paralysis, long-term follow-up after Artecoll^®^ injection laryngoplasty demonstrated sustained improvement in both subjective and objective voice outcomes for at least one year, with no reported adverse events. Furthermore, the therapeutic effect persisted for two years or longer [73]. Although sustained voice improvement has been reported, overcorrection or malposition is difficult to revise due to its permanence [73]. PDMS provides multi-year durability and stable augmentation; however, complications such as foreign-body reactions and extrusion have limited its current use [74,75,76]. The long-lasting effect of particulate synthetic materials is mediated by particle persistence and progressive encapsulation by host collagen. Accurate placement in a deep plane (intramuscular or paraglottic) can reduce the need for reinjection. However, as encapsulation progresses, reversibility decreases, making correction more difficult [68,72,73,75]. This contrasts with natural matrices such as HA, collagen, and fibrin, which undergo enzyme-mediated degradation and therefore have a time-limited efficacy [77,78,79].

In recent years, research has progressed beyond simple volumetric augmentation toward hybrid material designs that aim to achieve both mechanical stability and biological integration. The silk–HA composite combines the viscoelasticity of HA with the structural reinforcement provided by silk protein microparticles approximately 390 μm in size. Gradual degradation of silk contributes to mechanical stability, while HA preserves the elasticity required for vocal fold vibration. Beyond silk–HA hybrids, synthetic materials such as click-chemistry polyethylene glycol (PEG) hydrogels have been engineered to fine-tune elastic modulus and stress relaxation while enhancing dispersion. In preclinical laryngeal models, these materials demonstrated more homogeneous distribution, required lower injection pressures, and elicited less inflammation compared to CaHA [80,81]. From a viscoelastic standpoint, the clinical goal is to preserve mucosal wave propagation by approximating the low-modulus, high-frequency behavior of the SLP. The target viscoelastic modulus is approximately 100–200 Pa [82]. Particulate synthetic materials are advantageous for bulk volume but can behave as effectively stiffer at the injection plane, especially with superficial placement, which increases the risk of wave damping. HA-based systems can be tuned to physiological moduli and exhibit favorable shear-thinning properties during injection. Nevertheless, superficial bolus deposition and overfilling with HA can impair wave propagation, underscoring the critical importance of injection plane and dosage for both material classes [59,64,65]. More recently, surface and nanocomposite stabilization strategies have been proposed to reduce filler migration and heterogeneous dispersion. Examples include the use of polydopamine-coated PDMS to enhance tissue adhesion [83] and carbon-nanotube-reinforced hydrogels that improve mechanical strength and restore viscoelastic behavior [84].

These hybrid and polymer-based systems represent a shift from conventional fillers to next-generation injectables that integrate biological compatibility with regenerative potential, thereby laying the foundation for the regenerative injectable biomaterials discussed in the following section.

## 5. Injectable Materials for Vocal Fold Scarring; Tissue Regeneration

Irreversible lesions of the vocal fold such as scarring, atrophy, and sulcus cannot be adequately managed by volume augmentation alone and necessitate injectable approaches that promote genuine tissue regeneration. Contemporary formulations are designed not only to restore bulk but also to attenuate fibrotic remodeling, re-establish ECM architecture, and enhance viscoelastic properties essential for normal vibration [51]. Achieving functional regeneration requires not only material optimization but also the biological coordination of multiple elements. According to the principles of tissue engineering, this process depends on the integrated interaction of cells, scaffolds, and regulatory factors within appropriate biological conditions [85]. The following sections outline the key components of this regenerative strategy: growth factors, mesenchymal stem cells, and naturally derived injectable scaffolds, which collectively enable the functional restoration of the vocal fold.

### 5.1. Growth Factor/Drug Delivery

Vocal fold scarring is characterized by excessive deposition of disorganized collagen and loss of viscoelasticity, ultimately impairing phonatory function. Since fibrosis is largely mediated by dysregulated signaling pathways, particularly transforming growth factor-β (TGF-β), numerous studies have investigated the use of growth factors and pharmacologic agents to restore ECM integrity and promote tissue regeneration.

Among these, basic fibroblast growth factor (bFGF) and hepatocyte growth factor (HGF) play central roles. bFGF enhances fibroblast proliferation and HA synthesis while reducing type I collagen expression, thereby promoting balanced ECM remodeling. HGF exerts strong antifibrotic effects by suppressing TGF-β signaling and collagen accumulation, while also promoting angiogenesis and epithelial recovery [86].

Given the central profibrotic role of TGF-β1, approaches to modulate its downstream mediators such as SMAD3 have been investigated to attenuate scar formation [87,88]. Pharmacologic modulation has also been studied. Corticosteroids remain the most widely used anti-inflammatory adjunct, and antifibrotic agents such as pirfenidone, a small-molecule inhibitor of TGF-β/SMAD signaling, have shown promise in preclinical models by reducing collagen accumulation [89].

These findings suggest that the regenerative potential of growth factor–based therapy depends primarily on the ability to rebalance ECM synthesis and degradation while modulating the profibrotic signaling cascade. Sustained and localized delivery systems will further enhance their therapeutic effectiveness.

### 5.2. Mesenchymal Stem Cell

Research on cell-based therapy for vocal fold regeneration has focused on mesenchymal stem cells (MSCs) due to their potent paracrine and antifibrotic properties. Early studies utilized autologous bone marrow-derived MSCs (BM-MSCs), which reduced collagen deposition and improved viscoelasticity in canine and rabbit models of chronic vocal fold scarring [85]. Although some reports suggested that transplanted MSCs might contribute to tissue repair through differentiation and engraftment, subsequent studies demonstrated that most cells disappeared within four weeks [90,91]. This finding indicates that their therapeutic benefit is primarily mediated by the early secretion of growth factors and cytokines rather than by long-term cell survival.

Adipose-derived stem cells (ADSCs) offer an easily accessible autologous cell source and were first applied to vocal fold repair in a canine vocal fold injury model, where autologous ADSC injection combined with atelocollagen suggested potential for reducing fibrosis and promoting structural recovery [92]. Hiwatashi et al. demonstrated that both ADSCs and BM-MSCs produce comparable antifibrotic and ECM-restorative effects [93]. More recently, the adipose-derived stromal vascular fraction (ADSVF), a freshly isolated mixture containing MSCs along with endothelial and immune cell subsets, has gained attention as a one-step autologous graft. ADSVF exhibits similar antifibrotic potential without requiring culture expansion [94].

Apart from exogenous transplantation, endogenous MSC-like populations have been identified within the macula flava of the human vocal fold, suggesting the presence of an intrinsic stem cell niche that may contribute to tissue maintenance and spontaneous repair [95,96].

The outcome of stem cell therapy depends significantly on the delivery vehicle. Viscous or hydrogel-based carriers such as HA, fibrin, or collagen reduce shear stress–related cell injury during injection and enhance cell survival and paracrine signaling after implantation [97]. In vivo, small intestinal submucosa (SIS) scaffolds combined with MSCs have supported mucosal remodeling and improved vibratory function [98]. Additionally, encapsulating ADSCs within HA-alginate hydrogels further enhanced mucosal vibration and ECM reconstruction [99].

More recently, cell-free strategies utilizing MSC-derived secretomes or extracellular vesicles have emerged as promising alternatives. These acellular systems deliver growth factors, cytokines, and exosomes that replicate the paracrine benefits of MSCs while avoiding concerns related to cell manipulation, immune rejection, and tumorigenic transformation [100,101].

Collectively, both experimental and early clinical evidence indicate that MSC-based therapy provides a biologically safe and effective approach to restoring vocal fold vibration by suppressing fibrosis, reconstructing the ECM, and establishing a regenerative micro-environment within the lamina propria.

### 5.3. Naturally Derived Injectable Material

Naturally derived biomaterials possess intrinsic biocompatibility and structural similarity to the ECM, making them suitable as injectable scaffolds for treating vocal fold scarring. Unlike growth factor or drug delivery systems, which primarily serve as carriers for bioactive molecules, these materials can directly influence the wound environment and support endogenous tissue repair.

HA is the most extensively studied natural injectable for vocal fold repair. As the principal glycosaminoglycan of the lamina propria, it supports hydration, elasticity, and ECM organization. Within the vibratory layer, native HA provides a permissive environment that promotes fibroblast migration and orderly matrix restoration. Chemical modifications enable control over degradation, gelation, and bioactivity while preserving syringe-based injectability. Crosslinked formulations, such as HA–3,3′-dithiobis(propionohydrazide) (DTPH) and hybrid HA–PEG diacrylate/DTPH hydrogels, allow tuning of network density and in vivo residence time [102]. Other derivatives, including carboxymethylated HA (CMHA-S) and HA–gelatin copolymers (Extracel, HyStem-C), have demonstrated favorable preclinical outcomes, including reduced collagen deposition, improved lamina propria architecture, and restoration of mucosal vibration compared to untreated controls [103]. Overall, HA-based hydrogels serve as biocompatible and bioactive scaffolds that maintain viscoelasticity within the physiological range of the vocal fold, support balanced ECM remodeling, and help restore structural continuity while limiting fibrosis [46].

Collagen-based hydrogels have been investigated as biologically active scaffolds for vocal fold regeneration, as collagen constitutes more than 40% of the lamina propria proteome and provides tensile strength that counterbalances the elastic recoil generated by elastin. However, following injury, excessive upregulation of procollagen types I and III leads to a disorganized fibrillar architecture and increased tissue stiffness, underscoring the importance of controlled collagen remodeling [104]. Injectable collagen gels exhibit excellent biocompatibility and minimal immunogenicity, but their rapid biodegradation and the risk of impairing mucosal wave propagation when injected too superficially remain significant limitations [64]. To address these challenges, cross-linking strategies and composite matrices such as collagen–alginate and collagen–HA systems have been developed, demonstrating improved mechanical stability and enhanced ECM synthesis without inducing matrix contraction [105].

Beyond collagen, fibrin hydrogels represent another naturally derived platform with inherent hemostatic, anti-inflammatory, and antifibrotic properties. Fibrin scaffolds facilitate cell adhesion and ECM remodeling, yet their rapid degradation often necessitates cross-linking or hybridization with polymers such as dextran-MA to achieve mechanical stability [106].

Decellularized ECM (dECM) hydrogels represent one of the most tissue-specific classes of naturally derived injectable scaffolds for vocal fold regeneration. Unlike single-component polymers such as collagen or HA, dECM retains the complex biochemical composition of the vocal fold lamina propria, including collagens I, III, V, VI, XII, XIV, elastin, fibronectin, versican, decorin, and sulfated glycosaminoglycans (sGAGs). This composition provides microenvironmental cues that closely resemble those regulating fibroblast behavior, epithelial migration, and ECM homeostasis [44]. Proteomic analyses of bovine and porcine vocal fold ECMs have revealed the retention of more than 250 matrix-associated proteins and abundant sGAG content even after decellularization, indicating a broader biochemical profile compared with conventional collagen or HA gels. ECM-derived macromolecules and matrix-bound vesicles have been shown to downregulate α-SMA and COL1A1 expression in TGF-β1–stimulated fibroblasts, suggesting an inherent antifibrotic potential. Solubilized vocal fold ECM hydrogels exhibit viscoelastic moduli of approximately 100–200 Pa, closely matching those of the SLP, and their collagen–elastin architecture provides mechanical properties compatible with mucosal-wave vibration [82]. Translationally, intramucosal delivery aimed at remodeling SLP scarring remains in the preclinical stage; formulations will need to have viscoelastic properties and degradation kinetics specifically tuned to this low-modulus, high-frequency environment [53]. To address these challenges, composite dECM-polymer hydrogels and click chemistry–based in situ gelation techniques are being developed to enhance mechanical stability and residence time while preserving essential biochemical cues [107,108]. Emerging studies combine dECM scaffolds with stem cell–derived secretomes or extracellular vesicles to create acellular, bioinstructive injectable therapies [109,110]. In the airway, tracheal dECM/GelMA-nanoclay composites loaded with endothelial progenitor cell exosomes accelerated vascularized tissue repair in vivo [111]. Collectively, dECM hydrogels act not merely as inert fillers but as biologically active matrices that may modulate the wound microenvironment and promote functional tissue regeneration.

Overall, naturally derived injectable scaffolds function not only as passive fillers but also as bioactive matrices that actively engage in tissue repair. Their clinical potential lies in their ability to restore the viscoelastic and structural properties of the scarred vocal fold while minimizing adverse immune responses. Future studies should focus on optimizing their mechanical properties, degradation profiles, and integration with host tissue to achieve durable functional recovery.

### 5.4. Vocal Fold Mucosa Replacement

Beyond restoring the lamina propria, regenerative research has advanced toward reconstructing the entire epithelial–mucosal interface essential for vocal fold vibration. Chhetri et al. provided one of the earliest in vivo demonstrations of cell-based lamina propria regeneration using autologous cultured fibroblasts in a canine model, introducing the concept of lamina propria replacement therapy [112].

Ling et al. subsequently engineered a biofabricated human vocal fold mucosa composed of primary epithelial cells and fibroblasts. In an ex vivo canine larynx, the construct generated mucosal wave vibrations and acoustic outputs approaching normal patterns and survived without immune rejection in humanized mice. This established a functional proof of concept that human-derived cells can reconstitute a physiologically vibratory mucosa [113].

Fukahori et al. advanced this concept toward clinical application by creating a tissue-engineered mucosal construct using autologous oral epithelial cells and fibroblasts cultured on oriented collagen sheets. When transplanted onto mucosa-deficient canine vocal folds, the graft developed a stratified epithelium with fibroblast infiltration resembling the native lamina propria and partially restored mucosal wave motion, with reduced fibrosis compared to untreated controls [114].

Lungova et al. generated a three-dimensional vocal fold mucosa from human induced pluripotent stem cells (hiPSCs) by directing their differentiation toward vocal fold epithelial progenitors. Co-culture with primary fibroblasts on collagen scaffolds produced a stratified squamous epithelium expressing keratins (K13, K14, p63) and mucins (MUC1, MUC4), and exhibited smoke-induced inflammatory responses resembling those of native tissue. Although limited to in vitro modeling, this study established a developmentally guided, patient-specific platform for vocal fold mucosal regeneration [115].

Grossmann et al. developed an organotypic laryngeal mucosa model using buccal epithelial cells and immortalized human vocal fold fibroblasts, resulting in a multilayered epithelium with basement membrane characteristics and a proteomic profile closely resembling native tissue [116]. Hamilton et al. described a dehydrated collagen matrix embedded with umbilical cord–derived MSC (Cellogen) that released reparative cytokines and ECM proteins while reducing inflammation, suggesting its potential application for fibrotic mucosal replacement [117].

Recent work has introduced an in situ continuum robotic bioprinter that deposits a dopamine-grafted HA/silk fibroin hydrogel directly onto vocal fold defects using a laryngoscopic approach. In benchtop and ex vivo laryngeal models, the system achieved conformal restoration of defect geometry and produced smooth, contiguous prints on the vibrating surface. This approach addresses two persistent challenges in mucosal repair, namely on-surface retention in a wet, high-shear environment and precise placement on a thin, low-modulus cover. Although these results demonstrate technical feasibility, in vivo studies are necessary to confirm long-term vibratory durability, epithelial integration, and biocompatibility. Collectively, the data support 3D bioprinting as an emerging strategy for creating patient-specific mucosal patches that complement bioactive injectable therapies [118].

Together, these investigations illustrate a gradual transition from injectable or partial-layer therapies to complete mucosal reconstruction, highlighting the coordinated regeneration of the epithelium and lamina propria as the foundation for restoring vibratory pliability and phonatory function.

## 6. Preclinical and Clinical Application

### 6.1. Anti-Fibrotic and ECM-Restorative Therapies

Anti-fibrotic and ECM-restorative approaches aim to reduce excessive collagen deposition and recover the viscoelastic vibratory cover. bFGF has been most widely investigated. In the animal study, a single injection into scarred or atrophic lamina propria improved aerodynamic and acoustic parameters and restored mucosal-wave amplitude; no hypertrophic scarring was reported during the periods observed [119]. In small clinical series involving mainly atrophic or scarred vocal folds, and in a few cases sulcus, subepithelial bFGF injection improved aerodynamic, acoustic, and stroboscopic outcomes that were sustained for up to twelve months [119,120]. No treatment-related serious adverse events were reported within that timeframe, although use in patients with a history of neoplasia is generally avoided because of the theoretical risk of growth-factor-stimulated proliferation. In a separate clinical study, regenerative phonosurgery using a gelatin sponge impregnated with bFGF created a submucosal space within the scarred fold and enabled localized sustained release of the factor. This approach resulted in significant improvement in maximum phonation time, Voice Handicap Index(VHI)-10, and GRBAS (grade, roughness, breathiness, asthenia, strain) scores in a cohort of fifteen patients with vocal-fold scar or sulcus who received either direct bFGF injection or gelatin-bFGF implantation [121]. A small prospective series (*n* = 6) reported durable improvements in acoustic parameters and VHI-10 scores at least two years after treatment [122]. Regarding retreatment, many bFGF protocols consist of four weekly sessions; some indications (e.g., bowing or atrophy) have responded to single-session dosing. However, standardized criteria for maintenance or repeat courses beyond the initial series are lacking, and real-world retreatment rates are rarely reported in scar or sulcus cohorts [120,122,123]. Across clinical series of intracordal bFGF, serious drug-related events have not been reported. Typical minor events include transient hyperemia and roughness, and meta-analytic summaries similarly note no major adverse events [123,124]. A dedicated safety comparison suggests that trafermin (recombinant bFGF) is at least as safe as steroid injection in the early post-procedure period [125].

HGF has demonstrated anti-fibrotic and angiogenic activity in rabbit lamina-propria scar models by up-regulating HA synthase and matrix metalloproteinases and suppressing collagen-I deposition. Small phase I and II clinical studies in patients with scar or sulcus reported improvement in VHI and GRBAS scores, with no serious adverse events observed during sixth month of follow-up. Clinical dosing has involved a four-week course of weekly injections, with doses ranging from 1 to 10 μg per fold [126]. Beyond direct injection, sustained-release systems incorporating HGF, such as diblock copolymer formed from PEG and polycaprolactone (PCL) {MPEG-b-(PCL-ran-PLLA)} and small intestinal submucosa gels, have demonstrated prolonged local retention and enhanced mucosal regeneration in animal models [127,128]. However, human evidence currently extends only up to six months, and studies have not established long-term safety (beyond 24 months), defined re-injection frequency, or characterized immunogenicity, including anti-drug antibody responses.

Platelet-rich plasma (PRP), an autologous concentrate containing platelet-derived and vascular endothelial growth factors as well as TGF-β, has been applied as a biologic adjunct to enhance angiogenesis and ECM repair. Clinical injections in mild to moderate scarring, atrophy, and sulcus showed both short-term improvement in VHI scores and in stroboscopic vibration indices, with good short-term tolerance [129].

Pirfenidone, an anti-fibrotic agent already approved for idiopathic pulmonary fibrosis, reduced collagen-I and α-smooth-muscle-actin expression and fibroblast proliferation when delivered in a sustained-release formulation to ferret vocal-fold scars, but has not yet been tested in the larynx clinically [89].

Physical or energy-based ECM-remodeling approaches include angiolytic lasers and injectable nanofiber scaffolds. In animal scar models, pulsed-dye, potassium-titanyl-phosphate, and 532-nm diode lasers induced selective photothermolysis, modified ECM proteins, and reduced inflammatory cytokine expression; the diode laser also increased HA synthase and matrix metalloproteinase levels [130]. A pilot clinical study using pulsed-dye laser treatment in eleven patients with vocal-fold scarring reported significant short-term improvements in VHI, mucosal-wave amplitude, and mean phonatory flow without major adverse events [131].

A collagen–hyaluronic-acid nanofiber spray, tested in a rabbit model of lamina-propria trauma, reduced collagen-fiber diameter and suggested a reduction in scarring, but has not yet been evaluated in humans [132].

Beyond these approaches, ECM-mimetic hydrogel systems have emerged as promising injectable scaffolds for the functional restoration of the scarred lamina propria. Silk-HA composite hydrogels, developed by Brown et al. as injectable silk-protein microparticle–based fillers, exhibit tunable viscoelasticity within the physiological range of the vocal fold cover and maintain over 90% elastic recovery after compression. In porcine vocal fold models, injection of the silk-HA composite increased tissue stiffness by only about 20% immediately after delivery, thereby preserving native vibratory compliance. In vivo studies demonstrated slow degradation, biocompatibility, and host–cell infiltration. Early comparative data suggest superior volume stability and minimal migration relative to CaHA–CMC carriers, supporting its potential as a long-lasting yet ECM-friendly augmentation scaffold [133]. A recent retrospective clinical study of Silk-HA injection augmentation in 160 patients demonstrated a favorable safety profile, with hemilaryngeal edema occurring in 3.8% of cases and only 1.3% requiring hospitalization. Significant improvements in VHI-10 and CAPE-V scores were observed at all follow-up intervals, and approximately 27% of unilateral paralysis cases required re-medialization. While many patients maintained satisfactory phonatory outcomes beyond one year, the ultimate duration of efficacy remains to be determined [8].

Similar concepts include decellularized ECM hydrogels derived from laryngeal or other connective tissues, which retain native biochemical cues to promote site-specific matrix remodeling and angiogenesis. These next-generation ECM-mimetic hydrogels represent a shift from purely mechanical bulking to biologically instructive augmentation designed to support organized matrix deposition and restoration of pliability.

### 6.2. Cell-Based Regenerative Therapies

Early efforts at cell-based regenerative approach involved the use of autologous fibroblasts harvested from the buccal mucosa. Small clinical series reported modest improvement in mucosal-wave motion and in VHI scores, and no notable adverse reactions were observed [134].

Later studies shifted attention to stem-cell–based approaches. ADSCs were initially evaluated in an acutely wounded canine model, where autologous ADSC injections combined with atelocollagen reduced atrophy and morphological irregularities of the treated fold at 24 weeks, suggesting a potential structural benefit [92]. In rabbit models of chronic vocal-fold scarring, bone-marrow-derived mesenchymal stem cells (BM-MSCs) reduced collagen accumulation, thinned the fibrotic mucosa, restored hyaluronic-acid content, and shifted the collagen profile toward the more compliant type III pattern. These effects appear to result largely from paracrine immunomodulatory activity rather than from long-term engraftment of the transplanted cells [135].

In rabbit models, subepithelial ADSVF delivery reduced collagen density and inflammatory cytokines while up-regulating HA-synthase and matrix-metalloproteinase activity, which led to greater vibratory amplitude of the folds [136]. In a small phase I feasibility study involving eight patients with chronic vocal-fold scarring, no treatment-related safety concerns were reported. Seven patients improved by at least 18 points in the VHI, and most showed better glottal closure and mucosal vibration on stroboscopic assessment [137].

Beyond cell injection alone, several groups have evaluated mesenchymal stem cell–laden hydrogels as carriers to enhance cell retention and modulate the wound microenvironment. In a rabbit model, an HA–alginate hydrogel carrying human adipose-derived MSCs reduced disorganized collagen I and increased local HGF activity. Viscoelastic parameters and the mucosal wave amplitude ratio improved compared to PBS scar controls, and ECM remodeling was more favorable than with MSCs alone [99]. A composite gel of small-intestinal submucosa with MSCs similarly promoted scar remodeling, exhibiting broader HA distribution, better-controlled collagen synthesis, and superior videokymographic vibratory amplitude relative to MSCs or matrix alone [98]. Following vocal fold scar excision in a chronic rabbit model, implantation of an MSC-based bioequivalent composed of a PEG–fibrin hydrogel significantly reduced scar thickness and restored lamina propria mechanics, achieving a Young’s modulus of 1.15 ± 0.25 kPa, which is comparable to that of intact tissue at 1.17 ± 0.45 kPa [138].

Not all carriers have been uniformly beneficial. In a large preclinical dosing study comparing BM-MSCs alone, a tunable hyaluronan hydrogel, and two cell-in-hydrogel conditions, persistent moderate-to-marked inflammatory infiltrates were observed in all hydrogel-injected groups. In contrast, the BM-MSC–only group exhibited the most favorable profile, including earlier resolution of viscoelastic abnormalities and a coordinated downregulation of profibrotic and inflammatory genes (e.g., COL1A2, COL3, FN1, TGF-β1, α-SMA, IL-1β, IL-17β, TNF) compared to injured controls. By 10 weeks, tissue viscoelasticity in all groups had converged toward uninjured levels [139].

A phase I/II study compared two delivery strategies following surgical scar release: injecting autologous BM-MSCs into the lamina propria and thyroarytenoid muscle either as a cell suspension or combined with a HA gel. At 12 months, both groups demonstrated overall improvement in the VHI and phonation threshold pressure (PTP). The cell suspension group showed greater benefit, with VHI scores decreasing from 78 to 57 and PTP from 6.0 to 3.85 cm H_2_O, significantly outperforming the MSC + HA group (between-group difference in PTP, *p* = 0.006). High-speed imaging and blinded assessments indicated improved vibratory function and glottal closure in the majority of participants. No treatment-related serious adverse events were reported [140].

### 6.3. Skeletal Muscle and Nerve Regenerative Therapies

Skeletal muscle regenerative approaches focus on recovery of the thyroarytenoid (TA) muscle. bFGF, which in the lamina propria acts as an anti-fibrotic factor, has also been explored for its myogenic effects in the thyroarytenoid muscle [141]. Early administration of bFGF in recurrent laryngeal nerve injured rat models increased the pool of satellite cells and the number of neuromuscular junctions and partially mitigated atrophy [142]. Some experiments indicated a benefit from higher dosing, whereas others found no clear dose–response effect; optimal timing and dosage remain undefined [143]. Controlled-release bFGF combined with autologous fascia implantation in denervated rat TA muscle increased muscle mass and vascular area. HGF and c-Met agonists have been shown to activate satellite cells and promote myofiber growth in animal models, with c-Met agonists exhibiting prolonged biological activity [144]. Bimagrumab, a human monoclonal antibody to activin receptor II, has accelerated recovery of thigh-muscle volume in human immobilization-atrophy models, but has not yet been tested in the larynx or in TA-muscle disease, and its role in laryngeal sarcopenia remains speculative [145].

Neural-regenerative strategies are at an earlier stage. bFGF has also been reported to increase acetylcholine-receptor clustering and axonal sprouting at neuromuscular junctions in similar animal studies [141,142]. A PRP-loaded nerve-guidance conduit (NGC) promoted Schwann-cell migration and proliferation, restored TA-muscle thickness close to normal, and showed histologic axonal bridging in a rabbit model of recurrent-laryngeal-nerve transection [146].

## 7. Conclusions

Restoring normal vocal fold vibration requires reestablishing biomechanical precision and a balanced lamina propria microenvironment. Early studies employing growth factor delivery, autologous cells, and ECM-based hydrogels have improved tissue pliability and reduced scarring; however, durability and reproducibility remain uncertain. Significant limitations persist with ECM-based strategies, particularly the incomplete control of fibrotic remodeling. Decellularization presents major challenges: harsh physical processing can distort microarchitecture; many preparations lack rigorous evaluation of immune compatibility and reseeding performance; and detergent protocols may leave residual DNA that complicates clinical approval [147,148]. Additionally, field-specific, vocal fold-tailored benchmarks for decellularization quality are lacking, which hinders translation [149]. Progress will depend on correlating material design variables with phonatory outcomes by integrating objective vibratory metrics, standardized rheology, and tightly controlled fabrication processes. Establishing these property-to-phonation relationships and manufacturing controls should enable injectable biomaterials to advance beyond bulk augmentation toward true tissue regeneration and restoration of the dynamic, viscoelastic behavior required for natural phonation.

## Figures and Tables

**Table 1:** Classification of vocal fold augmentation materials.

Clinical Status	Category	Material/Commercial Example	Duration of Effect	Advantages/Strengths	Limitations/Complications
Historical use	Early synthetic (xenograft)	Paraffin, Teflon™ (PTFE paste), Silicone (PDMS)	Permanent	Strong initial augmentation effect	Severe foreign-body reaction, fibrosis, granuloma, difficult removal
	Temporary gel (collagen-derived)	Gelatin (Gelfoam^®^)	4–6 wk	Safe, inexpensive, easy handling	Very short duration, low viscoelasticity
Declining use	Biologic (xenograft)	Bovine collagen (Zyplast^®^)	3–6 mo	ECM component, low immunogenicity	Hypersensitivity, viral risk, rapid resorption
	Biologic (homograft)	Cadaveric dermis (Cymetra^®^)	1–6 mo	Reduced foreign-body response, human-derived	Short duration, inconsistent results
Off-label clinical use	Biologic (autologous)	Fat injection/Autologous fascia	6–18 mo (variable)	Excellent biocompatibility, similar viscoelasticity to native tissue	Unpredictable resorption (fat), donor-site morbidity (fascia)
	Natural polymer (ECM-derived)	Hyaluronic acid (Hylaform^®^, Restylane^®^, Reviderm^®^)	4–12 mo	Excellent biocompatibility, rheological similarity to SLP	Rapid degradation in native form, repeat injection required
Limited use	Synthetic polymer (hydrophilic)	Polyacrylamide hydrogel (PAAG)	>24 mo	Stable augmentation, minimal resorption	Rare granuloma, foreign-body risk
	Synthetic polymer (microsphere)	Polymethylmethacrylate (PMMA; Artecoll^®^)	Permanent	Durable volume maintenance	Difficult revision if malpositioned
	Synthetic elastomer	Polydimethylsiloxane (PDMS)	Multi-year	Long-lasting stability	Extrusion, foreign-body reaction
FDA approved	Mineral filler (bioceramic)	Calcium hydroxyapatite (CaHA; Radiesse^®^, Prolaryn Plus^®^)	18–24 mo	Only FDA-approved long-term filler, high biocompatibility	Potential VF stiffness if over-injected
	Hybrid bio-synthetic composite	Silk–HA composite (Silk Voice^®^)	≤18 mo	Combines HA viscoelasticity and silk mechanical support	Occasional reinjection needed
Preclinical	Advanced synthetic hydrogels	Click-chemistry PEG hydrogel	6–12 mo (experimental)	Tunable viscoelasticity, low inflammation	Preclinical stage
		Polydopamine-coated PDMS	—	Enhanced tissue adhesion	Cytotoxicity risk at high dose
Experimental	Advanced synthetic hydrogels	Carbon-nanotube-reinforced hydrogel	—	Improved mechanical strength and viscoelastic recovery	Uncertain biodegradability

VF, vocal fold; SLP, superficial lamina propria.

## Data Availability

The data presented in this study are available on request from the corresponding author.

## References

[1] Mallur P.S., Rosen C.A. (2010). Vocal fold injection: Review of indications, techniques, and materials for augmentation. Clin. Exp. Otorhinolaryngol..

[2] Kumai Y. (2019). Pathophysiology of Fibrosis in the Vocal Fold: Current Research, Future Treatment Strategies, and Obstacles to Restoring Vocal Fold Pliability. Int. J. Mol. Sci..

[3] Hansen J.K., Thibeault S.L. (2006). Current understanding and review of the literature: Vocal fold scarring. J. Voice.

[4] Nakayama M., Ford C.N., Bless D.M. (1993). Teflon vocal fold augmentation: Failures and management in 28 cases. Otolaryngol. Head Neck Surg..

[5] Lodder W.L., Dikkers F.G. (2015). Comparison of voice outcome after vocal fold augmentation with fat or calcium hydroxylapatite. Laryngoscope.

[6] Chhetri D.K., Jahan-Parwar B., Hart S.D., Bhuta S.M., Berke G.S. (2004). Injection laryngoplasty with calcium hydroxylapatite gel implant in an in vivo canine model. Ann. Otol. Rhinol. Laryngol..

[7] Gao W.Z., Paoletti M.F., Bensoussan Y., Bhatt N.K., van der Woerd B., Shuman E.A., Grant N., O’Dell K., Johns M.M. (2024). Prospective >12 Months Outcomes After Vocal Fold Injection Medialization with Silk Microparticle-Hyaluronic Acid Material. Laryngoscope.

[8] Dwyer C.D., Kridgen S., Chiang S., Fein M., Forrester C., Gordon L., Roth D.F., Shin J.J., Winston J., Carroll T.L. (2024). Silk-Hyaluronic Acid for Vocal Fold Augmentation: Safety Profile and Long-Term Voice Outcomes. J. Voice.

[9] Jiang J., Lin E., Hanson D.G. (2000). Vocal fold physiology. Otolaryngol. Clin. N. Am..

[10] Teller S.S., Farran A.J., Xiao L., Jiao T., Duncan R.L., Clifton R.J., Jia X. (2012). High-frequency viscoelastic shear properties of vocal fold tissues: Implications for vocal fold tissue engineering. Tissue Eng. Part A.

[11] Haji T., Mori K., Omori K., Isshiki N. (1992). Mechanical properties of the vocal fold. Stress-strain studies. Acta Otolaryngol..

[12] Gray S.D., Pignatari S.S., Harding P. (1994). Morphologic ultrastructure of anchoring fibers in normal vocal fold basement membrane zone. J. Voice.

[13] Hirano M. (1974). Morphological structure of the vocal cord as a vibrator and its variations. Folia Phoniatr..

[14] Madruga de Melo E.C., Lemos M., Aragao Ximenes Filho J., Sennes L.U., Nascimento Saldiva P.H., Tsuji D.H. (2003). Distribution of collagen in the lamina propria of the human vocal fold. Laryngoscope.

[15] Kelleher J.E., Siegmund T., Du M., Naseri E., Chan R.W. (2013). Empirical measurements of biomechanical anisotropy of the human vocal fold lamina propria. Biomech. Model. Mechanobiol..

[16] Alipour F., Vigmostad S. (2012). Measurement of vocal folds elastic properties for continuum modeling. J. Voice.

[17] Zhang Z. (2019). Structural constitutive modeling of the anisotropic mechanical properties of human vocal fold lamina propria. J. Acoust. Soc. Am..

[18] Dailey S.H., Tateya I., Montequin D., Welham N.V., Goodyer E. (2009). Viscoelastic measurements of vocal folds using the linear skin rheometer. J. Voice.

[19] Jiao T., Farran A., Jia X., Clifton R.J. (2009). High Frequency Measurements of Viscoelastic Properties of Hydrogels for Vocal Fold Regeneration. Exp. Mech..

[20] Chan R.W. (2018). Nonlinear viscoelastic characterization of human vocal fold tissues under large-amplitude oscillatory shear (LAOS). J. Rheol..

[21] Hartnick C.J., Rehbar R., Prasad V. (2005). Development and maturation of the pediatric human vocal fold lamina propria. Laryngoscope.

[22] Rousseau B., Hirano S., Scheidt T.D., Welham N.V., Thibeault S.L., Chan R.W., Bless D.M. (2003). Characterization of vocal fold scarring in a canine model. Laryngoscope.

[23] Ma Y., Long J., Amin M.R., Branski R.C., Damrose E.J., Sung C.K., Achlatis S., Kearney A., Chhetri D.K. (2020). Autologous fibroblasts for vocal scars and age-related atrophy: A randomized clinical trial. Laryngoscope.

[24] Honjo I., Isshiki N. (1980). Laryngoscopic and voice characteristics of aged persons. Arch. Otolaryngol..

[25] Gray S.D., Titze I.R., Alipour F., Hammond T.H. (2000). Biomechanical and histologic observations of vocal fold fibrous proteins. Ann. Otol. Rhinol. Laryngol..

[26] Thibeault S.L., Gray S.D., Bless D.M., Chan R.W., Ford C.N. (2002). Histologic and rheologic characterization of vocal fold scarring. J. Voice.

[27] Jette M.E., Hayer S.D., Thibeault S.L. (2013). Characterization of human vocal fold fibroblasts derived from chronic scar. Laryngoscope.

[28] Sato K., Hirano M. (1998). Electron microscopic investigation of sulcus vocalis. Ann. Otol. Rhinol. Laryngol..

[29] Hirano M., Yoshida T., Tanaka S., Hibi S. (1990). Sulcus vocalis: Functional aspects. Ann. Otol. Rhinol. Laryngol..

[30] Lee A., Sulica L., Aylward A., Scognamiglio T. (2016). Sulcus vocalis: A new clinical paradigm based on a re-evaluation of histology. Laryngoscope.

[31] Nunes R.B., Behlau M., Nunes M.B., Paulino J.G. (2013). Clinical diagnosis and histological analysis of vocal nodules and polyps. Braz. J. Otorhinolaryngol..

[32] AlGhamdi M.A., Alghamdi L.N., AlQazenli M.K., Alrashid D.S., Bakhsh Z. (2025). Effectiveness of laryngeal reinnervation compared to medialization thyroplasty in the treatment of unilateral vocal fold paralysis: A systematic review and network meta-analysis. World J. Otorhinolaryngol. Head. Neck Surg..

[33] Lee M., Mau T., Sulica L. (2021). Patterns of Recurrence of Phonotraumatic Vocal Fold Lesions Suggest Distinct Mechanisms of Injury. Laryngoscope.

[34] Bastian R.W., Cummings C.W., Flint P.W., Haughey B.H., Richardson M.A., Robbins T.K., Schuller D.E., Thomas J.R. (2005). Benign Vocal Fold Mucosal Disorders. Cummings Otolaryngology—Head and Neck Surgery.

[35] Swain S.K. (2024). Vocal fold cyst: A narrative review. J. Clin. Sci. Res..

[36] Zhang H., Chen Y., Cao D., Li W., Jing Y., Zhong H., Liu H., Zhu X. (2021). Optical biopsy of laryngeal lesions using femtosecond multiphoton microscopy. Biomed. Opt. Express.

[37] Jimenez-Socha M., Dion G.R., Mora-Navarro C., Wang Z., Nolan M.W., Freytes D.O. (2025). Radiation-Induced Fibrosis in Head and Neck Cancer: Challenges and Future Therapeutic Strategies for Vocal Fold Treatments. Cancers.

[38] Johns M.M., Kolachala V., Berg E., Muller S., Creighton F.X., Branski R.C. (2012). Radiation fibrosis of the vocal fold: From man to mouse. Laryngoscope.

[39] Tanigami Y., Kawai Y., Kaba S., Uozumi R., Ohnishi H., Kita T., Omori K., Kishimoto Y. (2022). Establishment of a radiation-induced vocal fold fibrosis mouse model. Biochem. Biophys. Res. Commun..

[40] Ivey C.M. (2019). Vocal Fold Paresis. Otolaryngol. Clin. N. Am..

[41] Crumley R.L. (1994). Unilateral recurrent laryngeal nerve paralysis. J. Voice.

[42] Beton S., Yucel L., Basak H., Ciler Buyukatalay Z. (2022). The Elderly Voice: Mechanisms, Disorders and Treatment Methods. Turk. Arch. Otorhinolaryngol..

[43] Chen X., Thibeault S.L. (2008). Characteristics of age-related changes in cultured human vocal fold fibroblasts. Laryngoscope.

[44] Wrona E.A., Peng R., Amin M.R., Branski R.C., Freytes D.O. (2016). Extracellular Matrix for Vocal Fold Lamina Propria Replacement: A Review. Tissue Eng. Part B Rev..

[45] Chan R.W., Titze I.R. (1999). Viscoelastic shear properties of human vocal fold mucosa: Measurement methodology and empirical results. J. Acoust. Soc. Am..

[46] Gaston J., Thibeault S.L. (2013). Hyaluronic acid hydrogels for vocal fold wound healing. Biomatter.

[47] Wan-Chiew N., Baki M.M., Fauzi M.B., Lokanathan Y., Azman M. (2021). In Vitro Evaluation of Biomaterials for Vocal Fold Injection: A Systematic Review. Polymers.

[48] Kimura M., Mau T., Chan R.W. (2010). Viscoelastic properties of phonosurgical biomaterials at phonatory frequencies. Laryngoscope.

[49] Rosen C.A. (2000). Phonosurgical vocal fold injection: Procedures and materials. Otolaryngol. Clin. N. Am..

[50] Warren J.P., Coe R.H., Culbert M.P., Dixon A.R., Miles D.E., Mengoni M., Beales P.A., Wilcox R.K. (2024). Injectable peptide-glycosaminoglycan hydrogels for soft tissue repair: In vitro assessment for nucleus augmentation. Mater. Adv..

[51] Coburn P.T., Li X., Li J.Y., Kishimoto Y., Li-Jessen N.Y.K. (2022). Progress in Vocal Fold Regenerative Biomaterials: An Immunological Perspective. Adv. Nanobiomed Res..

[52] Nejati S., Mongeau L. (2024). In Vitro Investigation of Vocal Fold Cellular Response to Variations in Hydrogel Porosity and Elasticity. ACS Biomater. Sci. Eng..

[53] Brown M., Okuyama H., Yamashita M., Tabrizian M., Li-Jessen N.Y.K. (2025). Trends in Injectable Biomaterials for Vocal Fold Regeneration and Long-Term Augmentation. Tissue Eng. Part B Rev..

[54] Kwon T.K., Buckmire R. (2004). Injection laryngoplasty for management of unilateral vocal fold paralysis. Curr. Opin. Otolaryngol. Head Neck Surg..

[55] Brunings W. (1911). Über eine neue Behandlungsmethode der Rekurrenslahmung. Verhandlungen Der Dtsch. Laryngol. Ges..

[56] Arnold G.E. (1962). Vocal rehabilitation of paralytic dysphonia IX. Technique of intracordal injection. Arch. Otolaryngol..

[57] Ford C.N., Martin D.W., Warner T.F. (1984). Injectable collagen in laryngeal rehabilitation. Laryngoscope.

[58] Remacle M., Lawson G. (2007). Results with collagen injection into the vocal folds for medialization. Curr. Opin. Otolaryngol. Head Neck Surg..

[59] Chung S.M., Kim H.S., Park H.S. (2011). Review of Injection Laryngoplasty as Treatment of Voice Disorders. Ewha Med. J..

[60] Mikaelian D.O., Lowry L.D., Sataloff R.T. (1991). Lipoinjection for unilateral vocal cord paralysis. Laryngoscope.

[61] Prstacic R., Slipac J., Zivkovic Ivanovic T., Simic I., Babic E., Danic Hadzibegovic A. (2020). Autologous Fat Augmentation in the Treatment of Unilateral Vocal Fold Paralysis—A 15-year Experience in a Single Institution. Acta Clin. Croat..

[62] Rihkanen H. (1998). Vocal fold augmentation by injection of autologous fascia. Laryngoscope.

[63] Schramm V.L., May M., Lavorato A.S. (1978). Gelfoam paste injection for vocal cord paralysis: Temporary rehabilitation of glottic incompetence. Laryngoscope.

[64] Kwon T.-K. (2006). Injection Laryngoplasty. Korean J. Otolaryngol.-Head. Neck Surg..

[65] Homicz M.R., Watson D. (2004). Review of injectable materials for soft tissue augmentation. Facial Plast. Surg..

[66] Rosen C.A., Thekdi A.A. (2004). Vocal fold augmentation with injectable calcium hydroxylapatite: Short-term results. J. Voice.

[67] Belafsky P.C., Postma G.N. (2004). Vocal fold augmentation with calcium hydroxylapatite. Otolaryngol. Head Neck Surg..

[68] Carroll T.L., Rosen C.A. (2011). Long-term results of calcium hydroxylapatite for vocal fold augmentation. Laryngoscope.

[69] Kharidia K.M., Bensoussan Y., Rosen C.A., Johns M.M., O’Dell K. (2023). Variations in Practices and Preferences of Vocal Fold Injection Materials: A National Survey. Laryngoscope.

[70] Lee S.W., Son Y.I., Kim C.H., Lee J.Y., Kim S.C., Koh Y.W. (2007). Voice outcomes of polyacrylamide hydrogel injection laryngoplasty. Laryngoscope.

[71] Kang K.Y., Lee S.A., Lee S.K., Seon S.W., Jung J.H., Park K.N., Kwak J.J., Lee S.W. (2016). Persistent Vocal Fold Granuloma Following Superficial PAAG Injection Laryngoplasty: A Case Report. J. Voice.

[72] Cohen S.R., Berner C.F., Busso M., Gleason M.C., Hamilton D., Holmes R.E., Romano J.J., Rullan P.P., Thaler M.P., Ubogy Z. (2006). ArteFill: A long-lasting injectable wrinkle filler material—Summary of the U.S. Food and Drug Administration trials and a progress report on 4- to 5-year outcomes. Plast. Reconstr. Surg..

[73] Min J.Y., Hong S.D., Kim K., Son Y.I. (2008). Long-term results of Artecoll injection laryngoplasty for patients with unilateral vocal fold motion impairment: Safety and clinical efficacy. Arch. Otolaryngol. Head Neck Surg..

[74] Sittel C., Thumfart W.F., Pototschnig C., Wittekindt C., Eckel H.E. (2000). Textured polydimethylsiloxane elastomers in the human larynx: Safety and efficiency of use. J. Biomed. Mater. Res..

[75] Sittel C., Echternach M., Federspil P.A., Plinkert P.K. (2006). Polydimethylsiloxane particles for permanent injection laryngoplasty. Ann. Otol. Rhinol. Laryngol..

[76] Bergamini G., Alicandri-Ciufelli M., Molteni G., Villari D., Luppi M.P., Genovese E., Presutti L. (2010). Therapy of unilateral vocal fold paralysis with polydimethylsiloxane injection laryngoplasty: Our experience. J. Voice.

[77] Parenteau-Bareil R., Gauvin R., Berthod F. (2010). Collagen-Based Biomaterials for Tissue Engineering Applications. Materials.

[78] Sall I., Férard G. (2007). Comparison of the sensitivity of 11 crosslinked hyaluronic acid gels to bovine testis hyaluronidase. Polym. Degrad. Stab..

[79] Sanz-Horta R., Matesanz A., Gallardo A., Reinecke H., Jorcano J.L., Acedo P., Velasco D., Elvira C. (2023). Technological advances in fibrin for tissue engineering. J. Tissue Eng..

[80] Kwon S., Choi H., Park C., Choi S., Kim E., Kim S.W., Kim C.S., Koo H. (2021). In vivo vocal fold augmentation using an injectable polyethylene glycol hydrogel based on click chemistry. Biomater. Sci..

[81] Gulka C.P., Brown J.E., Giordano J.E.M., Hickey J.E., Montero M.P., Hoang A., Carroll T.L. (2019). A novel silk-based vocal fold augmentation material: 6-month evaluation in a canine model. Laryngoscope.

[82] Mora-Navarro C., Badileanu A., Gracioso Martins A.M., Ozpinar E.W., Gaffney L., Huntress I., Harrell E., Enders J.R., Peng X., Branski R.C. (2020). Porcine Vocal Fold Lamina Propria-Derived Biomaterials Modulate TGF-beta1-Mediated Fibroblast Activation in Vitro. ACS Biomater. Sci. Eng..

[83] Chung E.J., Jun D.R., Kim D.W., Han M.J., Kwon T.K., Choi S.W., Kwon S.K. (2017). Prevention of polydimethylsiloxane microsphere migration using a mussel-inspired polydopamine coating for potential application in injection therapy. PLoS ONE.

[84] Ravanbakhsh H., Bao G., Latifi N., Mongeau L.G. (2019). Carbon nanotube composite hydrogels for vocal fold tissue engineering: Biocompatibility, rheology, and porosity. Mater. Sci. Eng. C Mater. Biol. Appl..

[85] Kanemaru S., Nakamura T., Omori K., Kojima H., Magrufov A., Hiratsuka Y., Hirano S., Ito J., Shimizu Y. (2003). Regeneration of the vocal fold using autologous mesenchymal stem cells. Ann. Otol. Rhinol. Laryngol..

[86] Ohno T., Hirano S., Rousseau B. (2009). Gene expression of transforming growth factor-beta1 and hepatocyte growth factor during wound healing of injured rat vocal fold. Laryngoscope.

[87] Paul B.C., Rafii B.Y., Gandonu S., Bing R., Born H., Amin M.R., Branski R.C. (2014). Smad3: An emerging target for vocal fold fibrosis. Laryngoscope.

[88] Hiwatashi N., Benedict P.A., Dion G.R., Bing R., Kraja I., Amin M.R., Branski R.C. (2017). SMAD3 expression and regulation of fibroplasia in vocal fold injury. Laryngoscope.

[89] Yamada T., Kumai Y., Kodama H., Nishimoto K., Miyamaru S., Onoue S., Orita Y. (2020). Effect of pirfenidone injection on ferret vocal fold scars: A preliminary in vivo study. Laryngoscope.

[90] Kanemaru S., Nakamura T., Yamashita M., Magrufov A., Kita T., Tamaki H., Tamura Y., Iguchi F., Kim T.S., Kishimoto M. (2005). Destiny of autologous bone marrow-derived stromal cells implanted in the vocal fold. Ann. Otol. Rhinol. Laryngol..

[91] Kim C.S., Choi H., Park K.C., Kim S.W., Sun D.I. (2018). The Ability of Human Nasal Inferior Turbinate-Derived Mesenchymal Stem Cells to Repair Vocal Fold Injuries. Otolaryngol. Head Neck Surg..

[92] Lee B.J., Wang S.G., Lee J.C., Jung J.S., Bae Y.C., Jeong H.J., Kim H.W., Lorenz R.R. (2006). The prevention of vocal fold scarring using autologous adipose tissue-derived stromal cells. Cells Tissues Organs.

[93] Hiwatashi N., Hirano S., Mizuta M., Tateya I., Kanemaru S., Nakamura T., Ito J. (2014). Adipose-derived stem cells versus bone marrow-derived stem cells for vocal fold regeneration. Laryngoscope.

[94] Mattei A., Magalon J., Bertrand B., Grimaud F., Revis J., Velier M., Veran J., Dessi P., Sabatier F., Giovanni A. (2018). Autologous adipose-derived stromal vascular fraction and scarred vocal folds: First clinical case report. Stem Cell Res. Ther..

[95] Sato K., Chitose S.I., Sato F., Sato K., Ono T., Umeno H. (2023). Vascularity in the macula flava of human vocal fold as a stem cell niche. Auris Nasus Larynx.

[96] Kurita T., Sato K., Chitose S., Fukahori M., Sueyoshi S., Umeno H. (2015). Origin of Vocal Fold Stellate Cells in the Human Macula Flava. Ann. Otol. Rhinol. Laryngol..

[97] Amer M.H., Rose F., Shakesheff K.M., White L.J. (2018). A biomaterials approach to influence stem cell fate in injectable cell-based therapies. Stem Cell Res. Ther..

[98] Choi J.W., Park J.K., Chang J.W., Kim D.Y., Kim M.S., Shin Y.S., Kim C.H. (2014). Small intestine submucosa and mesenchymal stem cells composite gel for scarless vocal fold regeneration. Biomaterials.

[99] Kim Y.M., Oh S.H., Choi J.S., Lee S., Ra J.C., Lee J.H., Lim J.Y. (2014). Adipose-derived stem cell-containing hyaluronic acid/alginate hydrogel improves vocal fold wound healing. Laryngoscope.

[100] Lee S.K., Lee S.C., Kim S.J. (2015). A novel cell-free strategy for promoting mouse liver regeneration: Utilization of a conditioned medium from adipose-derived stem cells. Hepatol. Int..

[101] Baglio S.R., Pegtel D.M., Baldini N. (2012). Mesenchymal stem cell secreted vesicles provide novel opportunities in (stem) cell-free therapy. Front. Physiol..

[102] Hansen J.K., Thibeault S.L., Walsh J.F., Shu X.Z., Prestwich G.D. (2005). In vivo engineering of the vocal fold extracellular matrix with injectable hyaluronic acid hydrogels: Early effects on tissue repair and biomechanics in a rabbit model. Ann. Otol. Rhinol. Laryngol..

[103] Thibeault S.L., Klemuk S.A., Chen X., Quinchia Johnson B.H. (2011). In Vivo engineering of the vocal fold ECM with injectable HA hydrogels-late effects on tissue repair and biomechanics in a rabbit model. J. Voice.

[104] Tang S.S., Mohad V., Gowda M., Thibeault S.L. (2017). Insights Into the Role of Collagen in Vocal Fold Health and Disease. J. Voice.

[105] Xu W., Hu R., Fan E., Han D. (2011). Adipose-derived mesenchymal stem cells in collagen-hyaluronic acid gel composite scaffolds for vocal fold regeneration. Ann. Otol. Rhinol. Laryngol..

[106] Li S., Dan X., Chen H., Li T., Liu B., Ju Y., Li Y., Lei L., Fan X. (2024). Developing fibrin-based biomaterials/scaffolds in tissue engineering. Bioact. Mater..

[107] Brown M., Okuyama H., Li L., Yang Z., Li J., Tabrizian M., Li-Jessen N.Y.K. (2026). Clicktetrazine dECM-alginate hydrogels for injectable, mechanically mimetic, and biologically active vocal fold biomaterials. Biomaterials.

[108] Mora-Navarro C., Smith E., Wang Z., Ramos-Alamo M.D.C., Collins L., Awad N., Cruz D.R.D., Tollison T.S., Huntress I., Gartling G. (2026). Injection of vocal fold lamina propria-derived hydrogels modulates fibrosis in injured vocal folds. Biomater. Adv..

[109] Wang G., Li Q., Liu S., Li M., Liu B., Zhao T., Liu B., Chen Z. (2024). An injectable decellularized extracellular matrix hydrogel with cortical neuron-derived exosomes enhances tissue repair following traumatic spinal cord injury. Mater. Today Bio.

[110] Liguori T.T.A., Liguori G.R., Sinkunas V., Correia C.J., Dos Santos Coutinho E.S.R., Zanoni F.L., Aiello V.D., Harmsen M.C., Moreira L.F.P. (2025). Intrapericardial injection of hydrogels with ASC and their secretome to treat dilated cardiomyopathies. Sci. Rep..

[111] Shen Z., Shan Y., Lu Y., Zhu J., Yuan L., Chen W., Sun F., Wang Q., Wang Y., Zhang Y. (2025). Decellularized extracellular matrix-loaded exosome hydrogel for cell-free tracheal scaffold in tracheal defect reconstruction and repair. J. Nanobiotechnol..

[112] Chhetri D.K., Head C., Revazova E., Hart S., Bhuta S., Berke G.S. (2004). Lamina propria replacement therapy with cultured autologous fibroblasts for vocal fold scars. Otolaryngol. Head Neck Surg..

[113] Ling C., Li Q., Brown M.E., Kishimoto Y., Toya Y., Devine E.E., Choi K.O., Nishimoto K., Norman I.G., Tsegyal T. (2015). Bioengineered vocal fold mucosa for voice restoration. Sci. Transl. Med..

[114] Fukahori M., Chitose S., Sato K., Sueyoshi S., Kurita T., Umeno H., Monden Y., Yamakawa R. (2016). Regeneration of Vocal Fold Mucosa Using Tissue-Engineered Structures with Oral Mucosal Cells. PLoS ONE.

[115] Lungova V., Chen X., Wang Z., Kendziorski C., Thibeault S.L. (2019). Human induced pluripotent stem cell-derived vocal fold mucosa mimics development and responses to smoke exposure. Nat. Commun..

[116] Grossmann T., Kirsch A., Grill M., Steffan B., Karbiener M., Brcic L., Darnhofer B., Birner-Gruenberger R., Gugatschka M. (2023). Introducing a new type of alternative laryngeal mucosa model. PLoS ONE.

[117] Hamilton N.J.I., Tait A., Weil B., Daniels J. (2024). The Use of a Dehydrated Cellularized Collagen Matrix to Replace Fibrotic Vocal Fold Mucosa. Laryngoscope.

[118] Groen S.A.T., Nejati S., AlHumaid S., Huynh L.A., Kost K., Sedal A., Mongeau L. (2025). A continuum robotic bioprinter for in situ vocal fold repair. Device.

[119] Ban M.J., Lee S.C., Park J.H., Park K.N., Kim H.K., Lee S.W. (2021). Regenerative efficacy of fibroblast growth factor for the treatment of aged vocal fold: From animal model to clinical application. Clin. Otolaryngol..

[120] Hirano S., Sugiyama Y., Kaneko M., Mukudai S., Fuse S., Hashimoto K. (2021). Intracordal Injection of Basic Fibroblast Growth Factor in 100 Cases of Vocal Fold Atrophy and Scar. Laryngoscope.

[121] Hirano S., Mizuta M., Kaneko M., Tateya I., Kanemaru S., Ito J. (2013). Regenerative phonosurgical treatments for vocal fold scar and sulcus with basic fibroblast growth factor. Laryngoscope.

[122] Sueyoshi S., Umeno H., Kurita T., Fukahori M., Chitose S.I. (2021). Long-term outcomes of basic fibroblast growth factor treatments in patients with vocal fold scarring, aged vocal fold, and sulcus vocalis. Auris Nasus Larynx.

[123] Kanazawa T., Komazawa D., Indo K., Akagi Y., Lee Y., Nakamura K., Matsushima K., Kunieda C., Misawa K., Nishino H. (2015). Single injection of basic fibroblast growth factor to treat severe vocal fold lesions and vocal fold paralysis. Laryngoscope.

[124] Hamilton N.J.I., Saccente-Kennedy B., Ambler G. (2023). The use of basic fibroblast growth factor to improve vocal function: A systematic review and meta-analysis. Clin. Otolaryngol..

[125] Hasegawa T., Fujita R., Komazawa D., Konomi U., Hirosaki M., Watanabe Y. (2025). Evaluation of Safety After Intracordal Basic Fibroblast Growth Factor Injection. J. Voice.

[126] Hirano S., Kawamoto A., Tateya I., Mizuta M., Kishimoto Y., Hiwatashi N., Kawai Y., Tsuji T., Suzuki R., Kaneko M. (2018). A phase I/II exploratory clinical trial for intracordal injection of recombinant hepatocyte growth factor for vocal fold scar and sulcus. J. Tissue Eng. Regen. Med..

[127] Choi J.S., Lee S., Kim D.Y., Kim Y.M., Kim M.S., Lim J.Y. (2015). Functional remodeling after vocal fold injury by small intestinal submucosa gel containing hepatocyte growth factor. Biomaterials.

[128] Choi J.W., Kim Y.S., Park J.K., Song E.H., Park J.H., Kim M.S., Shin Y.S., Kim C.H. (2017). Controlled Release of Hepatocyte Growth Factor from MPEG-b-(PCL-ran-PLLA) Diblock Copolymer for Improved Vocal Fold Regeneration. Macromol. Biosci..

[129] Woo P. (2023). Platelet-rich plasma in treatment of scar, atrophy, and sulcus: Short- and long-term results. Laryngoscope Investig. Otolaryngol..

[130] Kang H.T., Park K.N., Lee S.W. (2023). Regenerative Effect of a 532-nm Diode Laser on Vocal Fold Scar in a Rabbit Model. J. Voice.

[131] Mortensen M.M., Woo P., Ivey C., Thompson C., Carroll L., Altman K. (2008). The use of the pulse dye laser in the treatment of vocal fold scar: A preliminary study. Laryngoscope.

[132] Elibol E., Yilmaz Y.F., Unal A., Ozcan M., Kum N.Y., Kum R.O., Kulacoglu S. (2021). Effects of hyaluronic acid-collagen nanofibers on early wound healing in vocal cord trauma. Eur. Arch. Otorhinolaryngol..

[133] Brown J.E., Gulka C.P., Giordano J.E.M., Montero M.P., Hoang A., Carroll T.L. (2019). Injectable Silk Protein Microparticle-based Fillers: A Novel Material for Potential Use in Glottic Insufficiency. J. Voice.

[134] Chhetri D.K., Berke G.S. (2011). Injection of cultured autologous fibroblasts for human vocal fold scars. Laryngoscope.

[135] Svistushkin M.V., Kotova S.L., Shekhter A.B., Svistushkin V.M., Akovantseva A.A., Frolova A.A., Fayzullin A.L., Starostina S.V., Bezrukov E.A., Sukhanov R.B. (2019). Collagen fibrillar structures in vocal fold scarring and repair using stem cell therapy: A detailed histological, immunohistochemical and atomic force microscopy study. J. Microsc..

[136] Jeong J.Y., Park K.N., Lee S.W. (2023). A Novel Intervention That Prevents Vocal Fold Scarring. J. Voice.

[137] Mattei A., Bertrand B., Jouve E., Blaise T., Philandrianos C., Grimaud F., Giraudo L., Aboudou H., Dumoulin C., Arnaud L. (2020). Feasibility of First Injection of Autologous Adipose Tissue-Derived Stromal Vascular Fraction in Human Scarred Vocal Folds: A Nonrandomized Controlled Trial. JAMA Otolaryngol. Head Neck Surg..

[138] Svistushkin M., Shpichka A., Bikmulina P., Fayzullin A., Zolotova A., Kosheleva N., Selezneva L., Shavkuta B., Lobacheva V., Nikiforova A. (2023). Vocal fold restoration after scarring: Biocompatibility and efficacy of an MSC-based bioequivalent. Stem Cell Res. Ther..

[139] Bartlett R.S., Guille J.T., Chen X., Christensen M.B., Wang S.F., Thibeault S.L. (2016). Mesenchymal stromal cell injection promotes vocal fold scar repair without long-term engraftment. Cytotherapy.

[140] Hertegard S., Nagubothu S.R., Malmstrom E., LeBlanc K. (2020). Treatment of vocal fold scarring with autologous bone marrow-derived human mesenchymal stromal cells-first phase I/II human clinical study. Stem Cell Res. Ther..

[141] Goto T., Ueha R., Sato T., Yamasoba T. (2023). Effects of early local administration of high-dose bFGF on a recurrent laryngeal nerve injury model. J. Otolaryngol. Head Neck Surg..

[142] Kaneko M., Tsuji T., Kishimoto Y., Sugiyama Y., Nakamura T., Hirano S. (2018). Regenerative Effects of Basic Fibroblast Growth Factor on Restoration of Thyroarytenoid Muscle Atrophy Caused by Recurrent Laryngeal Nerve Transection. J. Voice.

[143] Goto T., Ueha R., Sato T., Fujimaki Y., Nito T., Yamasoba T. (2020). Single, high-dose local injection of bFGF improves thyroarytenoid muscle atrophy after paralysis. Laryngoscope.

[144] Choi H., Yu S.S., Choi J., Kim C.S. (2022). The Regenerative Effects of c-Met Agonistic Antibodies in Vocal Fold Atrophy. Int. J. Mol. Sci..

[145] Rooks D.S., Laurent D., Praestgaard J., Rasmussen S., Bartlett M., Tanko L.B. (2017). Effect of bimagrumab on thigh muscle volume and composition in men with casting-induced atrophy. J. Cachexia Sarcopenia Muscle.

[146] Kim J.W., Kim J.M., Choi M.E., Jeon E.J., Park J.M., Kim Y.M., Choi S.H., Eom T., Shim B.S., Choi J.S. (2022). Platelet-rich plasma loaded nerve guidance conduit as implantable biocompatible materials for recurrent laryngeal nerve regeneration. npj Regen. Med..

[147] Neishabouri A., Soltani Khaboushan A., Daghigh F., Kajbafzadeh A.M., Majidi Zolbin M. (2022). Decellularization in Tissue Engineering and Regenerative Medicine: Evaluation, Modification, and Application Methods. Front. Bioeng. Biotechnol..

[148] Gilbert T.W., Freund J.M., Badylak S.F. (2009). Quantification of DNA in biologic scaffold materials. J. Surg. Res..

[149] Mesina M., Mindrila I., Mesina-Botoran M.I., Mindrila L.A., Farhangee A., Pirici I. (2023). Optimization Techniques of Single-Detergent Based Protocols for Heart Tissue Decellularization. Curr. Health Sci. J..

